# Evolutions in Microstructure and Properties of Cu-Ni-Si-Mg-Mn Multi-Element High-Solute Alloy During a Short-Time Solid Solution Treatment, Aging, and Cold-Rolling

**DOI:** 10.3390/ma19081485

**Published:** 2026-04-08

**Authors:** Yuhang Zhang, Xiaolong Feng, Qingke Zhang, Xiang Lu, Cheng Xu, Xinli Zhang, Feng Liu, Zhenlun Song

**Affiliations:** 1 College of Materials Science and Engineering, Zhejiang University of Technology, Hangzhou 310014, China; zhangyuhang@nimte.ac.cn; 2 State Key Laboratory of Advanced Marine Materials, Ningbo Institute of Materials Technology and Engineering, Chinese Academy of Sciences, Ningbo 315201, China; luxiang@nimte.ac.cn (X.L.); xucheng@nimte.ac.cn (C.X.); songzhenlun@nimte.ac.cn (Z.S.); 3 Ningbo Kangqiang Electronics Co., Ltd., Ningbo 315105, China; fxl@kangqiang.com (X.F.); zhangxl@kangqiang.com (X.Z.); 4 Ningbo Xingye Shengtai Group Co., Ltd., Ningbo 315336, China; liuf@cn-shine.com

**Keywords:** high-solute alloy, multi-alloying, short-time solution, microstructure evolution, strength, electrical conductivity

## Abstract

**Highlights:**

- A multi-element CuNiSiMgMn high-solute alloy was designed to get ultrahigh strength.
- Recrystallization and precipitate decomposition behaviors at 1000 °C are revealed.
- A strip with a tensile strength of >950 MPa and a conductivity of >30% IACS was obtained.

**Abstract:**

To obtain ultrahigh strength Cu alloy strip for board-to-board connectors, a CuNiSiMgMn multi-element high-solute alloy was designed, and high-temperature short-time solid solution was utilized to optimize the properties of this alloy. The evolution in microstructure and properties of the cold-rolled CuNiSiMgMn alloy strip during high-temperature short-time solid solution, aging, and further cold-rolling are investigated. The results reveal that there are high-density Ni_x_Si precipitates and deformation defects in the original cold-rolled CuNiSiMgMn alloy strip. During a solid solution at 1000 °C, recrystallization primarily occurs between 15 and 30 s, while precipitate decomposition starts at a solid solution time of ~30 s and is almost complete 10 s later. With further increase in the solid solution time, the grain size of the alloy grows rapidly, but the residual precipitate particles exhibit little change. Upon aging at 500 °C for 2 h and a further 80% cold-rolling, nano-sized precipitates are formed, yielding high-strength alloy strips. The 80% cold-rolling increases the microhardness by 12% and decreases the electrical conductivity by 3% IACS. The strip solid solution-treated for 35 s exhibits the maximum strength, with a tensile strength of >950 MPa and a conductivity of >30% IACS. Further extension of the solid solution time decreases both the tensile strength and elongation. This work clarifies the critical time of recovery, recrystallization, and precipitate decomposition of the CuNiSiMgMn alloy during high-temperature solid solution and provides guidance for industrial production.

## 1. Introduction

The Cu-Ni-Si series alloys are widely used in the manufacture of electrical connectors, lead frames, and contact wires owing to their high strength, high electrical conductivity, good thermal conductivity, and relatively low cost [1,2,3]. As board-to-board (BTB) connectors keep developing toward higher density, a larger number of pins, narrower pitch, an ultralow height, and higher reliability, the performance requirements of the Cu alloy strip used for BTB connectors become increasingly stringent [4,5,6]. For a BTB micro connector with 48 terminals, the strength shall exceed 950 MPa, slightly higher than most of the existing Cu-Ni-Si series ternary alloys, and the required electrical conductivity exceeds 30% IACS.

Recently, two approaches have been employed to improve the strength of Cu-Ni-Si alloys. The first one involves the introduction of additional alloying elements, such as magnesium (Mg), chromium (Cr), cobalt (Co), and manganese (Mn) [7,8]. Among these alloy elements, the Mg element can effectively inhibit the growth of precipitates and the Mg atoms can pin dislocations. It was observed that the incorporation of Mg into a Cu-Ni-Si alloy can optimize the morphology and distribution of intragranular precipitates and exert a pinning effect on dislocations, which refine the grains and improve the strength and stress relaxation resistance of the alloy [9,10]. The Cr element can directly improve the electrical conductivity by forming Cr_3_Si intermetallic compounds (IMCs) in the alloy [11,12,13,14]. The Co atoms can substitute the Ni atoms to form (Ni,Co)_x_Si precipitates, and the addition of a small amount of Co can simultaneously optimize the mechanical and electrical properties of the alloy [15,16,17,18]. The addition of the Mn element leads to the formation of film-like IMCs along the grain boundaries (GBs), which can strongly improve the GB strength and thermal stability of the alloy [19,20]. In addition, the Mn elements considerably mitigate the hardness decline when the alloy is overaged. It should be noted that increasing the content of alloying elements can improve the strength of the alloy, but will also increase the difficulty of precipitate decomposition.

The second approach to improve the strength of the alloy focuses on optimizing the combination of deformation and heat treatment, primarily at the final processing steps, such as solid solution treatment, aging, and cold-rolling [21,22,23,24,25,26,27]. It was found that after a solid solution at 920 °C for 1 h and aging at 450 °C for 28 h, the Cu-Ni-Si alloy achieved a tensile strength of 788 MPa and an electrical conductivity of 45.0% IACS [28]. In another study, a Cu-Ni-Si-Co alloy was 60% cold-deformed, followed by aging at 500 °C for 2 h, and an electrical conductivity of 35.8% IACS and a tensile strength of 755 MPa were obtained [29]. To achieve sufficient solid solution, most existing studies have adopted a solid solution time of over 1 h, which is difficult to be used in the continuous annealing furnace. It has been revealed that the solid solution temperature rather than time is the dominate factor for precipitates decomposition, and high-temperature short-time solid solution can ensure sufficient decomposition without marked grain coarsening [30].

Based on the above reports, herein, the Mg and Mn elements are simultaneously added into the Cu-Ni-Si alloy to obtain a Cu-Ni-Si-Mg-Mn multi-element high-solute alloy. The total content of Ni and Si in the designed alloy is higher than that in the common commercial Cu-Ni-Si alloys such as the C70250 and C70350 alloys, and the co-addition of the Mn and Mg enables synergistic intragranular GB regulation of the microstructure, aiming to break through the strength limits of the existing commercial alloys. Considering studies on precipitate decomposition, recrystallization, and aging precipitation behaviors of Cu-Ni-Si series alloys with multi-element high-solute alloy remain scarce, it is crucial to reveal the precipitate decomposition and microstructure evolution behaviors of Cu-Ni-Si-Mg-Mn alloy strips to further optimize their performance. Therefore, a cold-rolled Cu-Ni-Si-Mg-Mn alloy was subjected to a short-time solution treatment at 1000 °C, followed by aging at 500 °C for 2 h, and finally an 80% cold-rolling. The evolution in grain structure, precipitates, tensile properties, fracture behavior, microhardness, and electrical conductivity were characterized throughout the process to offer insights into final processing of the Cu-Ni-Si-Mg-Mn multi-element high-solute alloy.

## 2. Materials and Methods

### 2.1. Specimen Preparation

**Figure 1:**
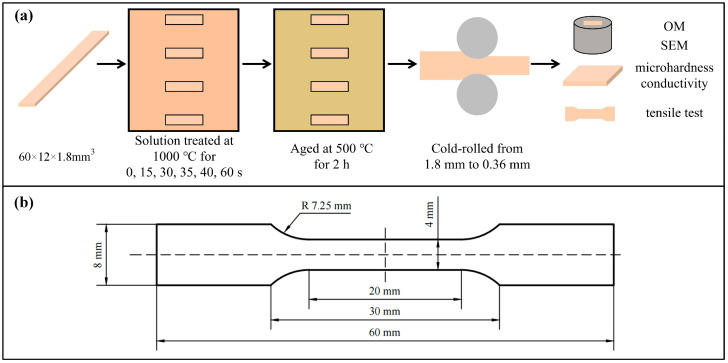
(**a**) Preparation and characterization process of the Cu-Ni-Si-Mg-Mn multi-element high-solute alloy specimens; (**b**) size of the tensile specimens.

The Cu-Ni-Si-Mg-Mn alloy strip used in this study was provided by Ningbo Xingye Shengtai Group Co., Ltd. (Ningbo, China), with a composition of 4.13% Ni, 0.73% Si, 0.17% Mg, and 0.14% Mn (wt.%), which was measured using inductively coupled plasma optical emission spectrometry (ICP-OES, SPECTRO ARCOS). The contents of Ni and Si follow the stoichiometric ratio of the δ-Ni_2_Si strengthening phase, with a Ni/Si atomic ratio of approximately 4:1. The contents of Mg and Mn were selected based on the effective microalloying ranges proposed in the literature [9,10,19,20]. An initial alloy plate with a thickness of 10.9 mm was obtained by hot-rolling of a homogenized ingot. Subsequently, the plate was rolled at room temperature to a final thickness of 1.8 mm, with a total cold-rolling reduction of ~83.5%. The cold-rolling process was conducted in 12 passes, with the reduction per pass controlled between 8~12% to prevent edge cracking. No intermediate annealing was performed throughout the whole cold-rolling process to preserve a high level of strain energy, which serves as the driving force for recrystallization during the subsequent solid solution. Specimens measuring 60 mm × 12 mm × 1.8 mm were cut via electrical discharge wire cutting (EDWC). These specimens were then solid solution-treated in a preheated box-type atmosphere furnace (HMX-1600-20) at 1000 °C for 15, 30, 35, 40, and 60 s, followed by water quenching. Subsequently, part of the quenched samples were aged in a muffle furnace at 500 °C for 2 h. A solid solution temperature of 1000 °C was selected to achieve rapid dissolution of precipitates based on previous findings [28]. The aging temperature of 500 °C for 2 h was chosen according to typical aging conditions for Cu-Ni-Si alloys [10]. Lastly, the aged samples were cold-rolled to a thickness of 0.36 mm. The complete specimen preparation process is illustrated in Figure 1a.

### 2.2. Microstructure Characterization

Small pieces were cut from specimens at different preparation stages via EDWC. The small pieces were mounted in epoxy resin for microstructural characterization. The specimens were first ground using graded sandpapers (from coarse to fine), then polished, and finally etched. The etching solution for the metallographic specimens was composed of ferric chloride (FeCl_3_, 5 g), hydrochloric acid (HCl, 10 mL), and anhydrous ethanol (100 mL). For scanning electron microscopy (SEM) observation, the specimens were etched using a 50% (*v*/*v*) aqua regia solution to expose the precipitates. The grain structures of the specimens were observed using a metallographic fluorescence microscope (NNM-800RF), and the grain size was calculated using the ImageJ v1.53a software. The morphologies and distribution of the precipitates were observed using a SEM (Zeiss Sigma 300) (Carl Zeiss, Oberkochen, Germany).

A transmission electron microscope (TEM) was used to reveal the precipitations after aging. To prepare the specimens for TEM observation, the alloy strip was solid solution-treated for 35 s, aged, and then cut into pieces of 12 × 12 × 1.8 mm using EDWC. Subsequently, the samples were finely ground until a thickness of ~50 µm was reached, and then they were punched into small disks with a diameter of 3 mm. Ion thinning was employed for the final processing of the TEM samples. The observations were conducted using a TEM (Talos F200X) (Thermo Fisher, Waltham, MA, USA) under an operating voltage of 200 kV, and the distribution of the elements was detected via energy-dispersive X-ray spectroscopy (EDS).

### 2.3. Property Tests

The preparation of the specimens for microhardness analysis followed the same procedure as that of the microstructural characterization. The Vickers microhardness was measured using a TMVS-1 microhardness tester (Beijing Times Lianchuang Technology Co., Ltd., Beijing, China) under a load of 200 g and a holding time of 15 s. Seven measurements were performed on each sample. The maximum and minimum values were discarded, and the average of the remaining microhardness values was calculated for each sample.

The tensile tests were conducted according to the Chinese GB/T 228.1-2021 standard [31]. For the tensile tests, dog bone-shaped specimens were prepared via EDWC; the shape and size of the tensile specimens are shown in Figure 1b. The edges of the specimens were ground to eliminate the machining marks, and tensile tests were conducted using an electronic universal testing machine (MTS/E45.105) under a crosshead speed of 1.0 mm/min. Three or more specimens were tested for each group, and the fracture morphologies of all the specimens were observed.

The electrical conductivities of the specimens were determined using a Sigma 2008 digital eddy current conductivity meter (Tianyan Instrument, Xiamen, China). Seven measurements were performed for each sample, and the average values were calculated after excluding the maximum and minimum values.

## 3. Results

### 3.1. Microstructure Evolution During Solid Solution and Aging

**Figure 2:**
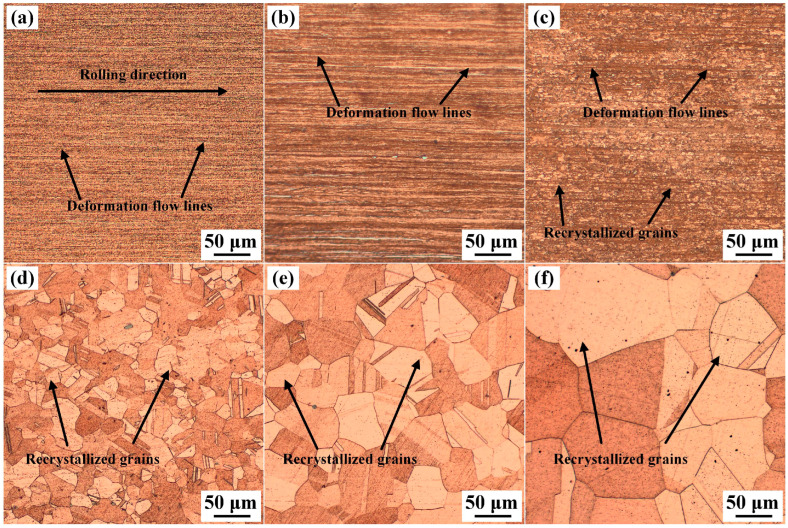
Metallographic images of the cold-rolled Cu-Ni-Si-Mg-Mn specimens solid solution-treated at 1000 °C for (**a**) 0 s, (**b**) 15 s, (**c**) 30 s, (**d**) 35 s, (**e**) 40 s, and (**f**) 60 s.

Figure 2 presents the metallographic images of the cold-rolled Cu-Ni-Si-Mg-Mn specimens solid solution-treated at 1000 °C for varying durations. As shown in Figure 2a, the original cold-rolled sample (before solid solution) exhibits a typical fibrous deformation texture. Owing to the compressive and shear forces applied during cold-rolling, the grains are elongated and flattened along the rolling direction. With the introduction of dislocations and stacking faults during the cold-rolling process [24], which become preferential corrosion sites, the GBs blur or even vanish after extreme cold-rolling. In addition, cold-rolling induces preferential grain alignment along the rolling direction, resulting in deformation flow lines parallel to the rolling direction. Figure 2b shows the metallographic microstructure of the specimen solid solution-treated for 15 s. Evidently, during the solid solution period of 0–15 s, partial recovery occurs, and the density of the deformation defects decrease, thus the deformation flow lines become wider, although the GBs are difficult to identify. When the solid solution time exceeds 30 s, recrystallization occurs, some fine equiaxed grains become visible (Figure 2c), and the density of the deformation flow lines decreases significantly. Immediately after 5 s, the recrystallized grains become apparent, and the grain size increases sharply (Figure 2d), with no residual deformation trace, indicating that recrystallization was achieved within a very short time at 1000 °C. Following solid solution for 40 and 60 s, the grain size markedly increased (Figure 2e,f). Quantitative statistical results of grain size show that the average grain size of the alloy reaches 25.72 μm after solid solution for 35 s, rapidly increases to 52.85 μm at 40 s, and further grows to 104.05 μm at 60 s. The observation of the metallographic structure confirms that during solid solution at 1000 °C, recrystallization of the cold-rolled Cu-Ni-Si-Mg-Mn alloy strip was initiated after 15 s, and recrystallization occurs mainly between 15 and 30 s, after which grain coarsening increases continuously with solid solution time.

**Figure 3:**
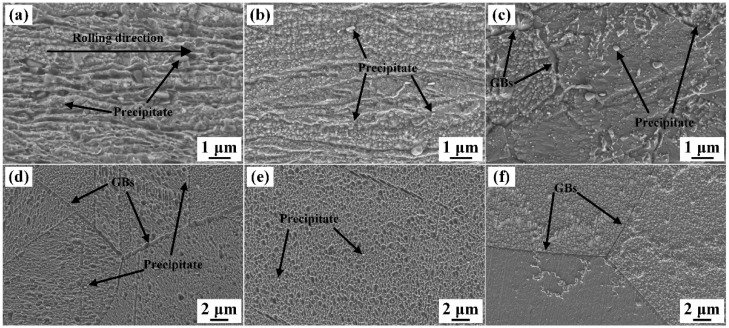
Microstructure of the cold-rolled Cu-Ni-Si-Mg-Mn specimens solid solution-treated at 1000 °C for (**a**) 0 s, (**b**) 15 s, (**c**) 30 s, (**d**) 35 s, (**e**) 40 s, and (**f**) 60 s.

The microstructure of the specimens solid solution-treated for different durations are shown in Figure 3. As presented in Figure 3a, the flow lines parallel to the rolling direction are obvious in the cold-rolled specimen, and there are high-density rod-like and spherical precipitate particles, most of which measure several hundred nanometers in size. Previous studies have revealed that the precipitates are mostly δ-Ni_2_Si IMCs [32,33]. Owing to the high density of deformation flow lines in the cold-rolled alloy, only a small number of precipitates are visible. Figure 3b shows the specimen solid solution-treated for 15 s, in which recovery has occurred, and the density of the flow lines decreases, making the precipitates clearer, but few recrystallized grains can be observed. Following solid solution for 30 s, fine equiaxed grains emerge, signaling a high degree of recrystallization, as shown in Figure 3c. Meanwhile, the precipitates are easier to be observed, while the density of the precipitation particles changes little compared with that shown in Figure 3a,b. After solid solution for 35 and 40 s, although some precipitation particles still exist, their density becomes extremely low, and their size is much smaller (Figure 3d,e). Meanwhile, few defects exist in the recrystallized grains. After 60 s, the precipitates are almost completely decomposed (Figure 3f). By comparing the metallographic images and SEM images, it can be predicted that recrystallization started before solid solution for 30 s and was completed within a very short time. By contrast, precipitate decomposition becomes notable after solution for over 30 s, while sufficient decomposition of the precipitates is achieved after solution for 40 s. Therefore, sufficient precipitate decomposition and grain coarsening shall be considered simultaneously.

**Figure 4:**
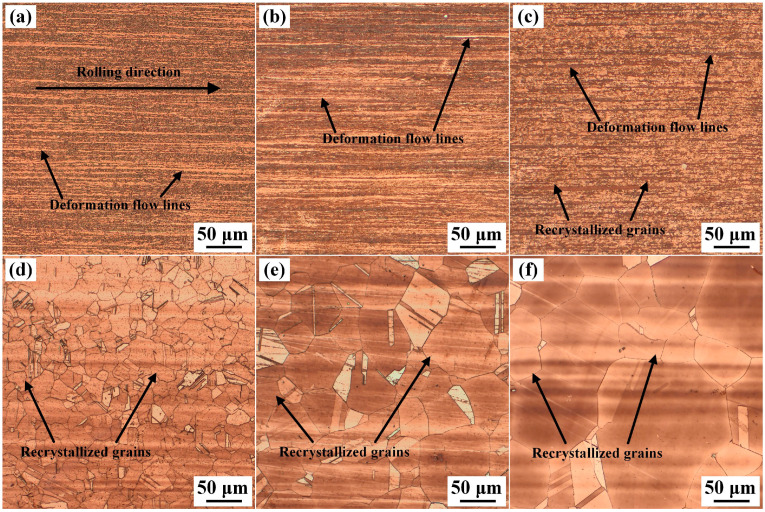
Metallographic images of the Cu-Ni-Si-Mg-Mn specimens aged at 500 °C for 2 h after being solid solution-treated at 1000 °C for (**a**) 0 s, (**b**) 15 s, (**c**) 30 s, (**d**) 35 s, (**e**) 40 s, and (**f**) 60 s.

The metallographic images of the samples solution-treated for different durations and then aged at 500 °C for 2 h are displayed in Figure 4. For the cold-rolled specimen, the density of the deformation defects is lower after aging, with some elongated grains visible along the rolling direction, as shown in Figure 4a, while the flow lines show little change, because recovery occurs in the cold-rolled strip at 500 °C and part of the deformation defects was eliminated. Since the precipitates formed during the aging process are ultrafine in size [30], they cannot be observed by metallographic observation. For the specimens annealed at 1000 °C, the effect of aging at 500 °C on the grain structure is negligible, thus the images shown in Figure 4b–f have no obvious differences from the non-aged samples.

**Figure 5:**
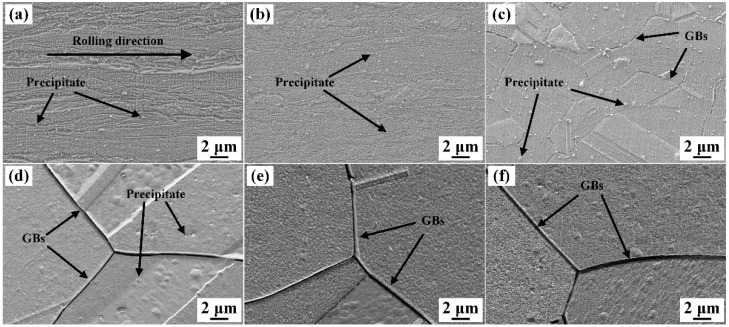
Microstructure of the Cu-Ni-Si-Mg-Mn specimens aged at 500 °C for 2 h after being solid solution-treated at 1000 °C for (**a**) 0 s, (**b**) 15 s, (**c**) 30 s, (**d**) 35 s, (**e**) 40 s and (**f**) 60 s.

Figure 5 shows the SEM images of the samples after solid solution and aging. It can be found that there are fewer defect-induced corrosion pits on the etched surfaces of the aged specimens. For the cold-rolled specimen with no solid solution, most of the deformation defects can be eliminated during the aging process, and even partial recrystallization may occur, making the etched surface much smoother, as shown in Figure 5a. Although most of the deformation defects were eliminated for the specimens solid solution-treated for different durations, there might be some residual defects, as the solid solution time are very short. The prolonged aging can further optimize the microstructure within the grains, resulting in etched surfaces with fewer surface irregularities (Figure 5b–f). Meanwhile, the residual precipitate particles that have not been decomposed will grow in size, making the particles easier to be observed, while the residual precipitates are very few for the specimens solid solution-treated for over 35 s.

**Figure 6:**
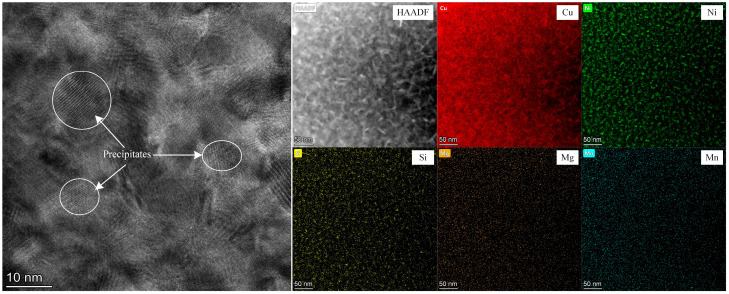
TEM micrographs and elements distribution of the Cu-Ni-Si-Mg-Mn specimens aged at 500 °C for 2 h after being solid solution-treated at 1000 °C for 35 s.

TEM was used to observe the precipitates formed during the aging process. As a representative, the TEM micrographs and the elemental distribution of the specimens aged at 500 °C for 2 h after solid solution-treated for 35 s are shown in Figure 6. The high-resolution image shows that precipitation particles of ~10 nm in size are formed in the specimen, and their density is relatively high. Meanwhile, the EDS mapping of the elements reveals the segregation of the Ni and Si elements, and the size of the segregation zones are comparable to that of the precipitation particles. On this basis, it can be predicted that Ni_x_Si precipitates are formed, although the exact atomic ratio of the precipitates can hardly be determined [10,11]. By contrast, the distributions of the Mg and Mn elements are uniform, demonstrating that these two elements exist as solute atoms.

### 3.2. Evolutions of Microhardness, Tensile Strength, and Fracture Behavior

**Figure 7:**
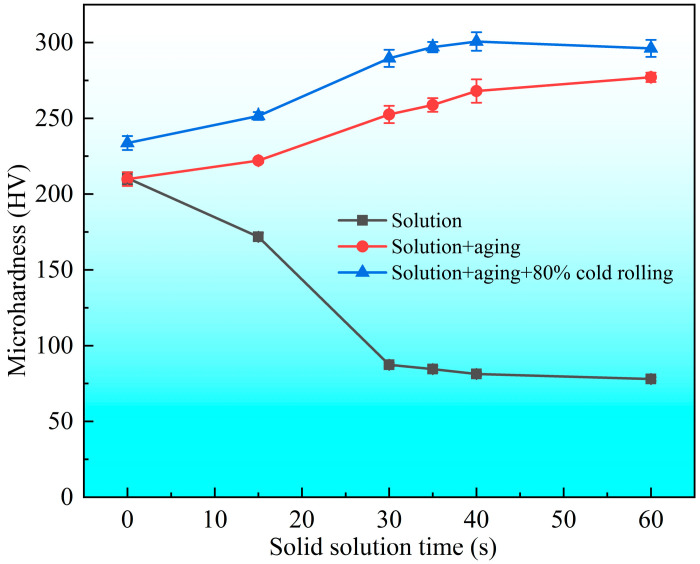
Variation in microhardness with solid solution time for the specimens of the post solution state, post solution + aging state, and post solution + aging + cold-rolling state.

Microhardness is an easily accessible parameter of the mechanical properties. Figure 7 illustrates the variation in microhardness with solid solution time for the specimens of three different states (solid-solution-treated, post-aging, and post-cold-rolling). It is observed that the microhardness of the solid solution-treated samples decreases rapidly as the solid solution time increases at the early stage, and then decreases slowly after the solid solution time exceeds 30 s. This is because the high-density dislocations induced by the prior cold-rolling are quickly eliminated during the solid solution process. Following recovery and recrystallization, the work hardening is completely removed, and the subsequent grain coarsening has minimal effect on the microhardness. Following the solid solution treatment and aging, the microhardness of the non-solution-treated sample exhibits minimal change compared with its pre-aging state owing to the elimination of work hardening and the occurrence of precipitation strengthening. By contrast, the specimens that underwent solid solution treatment (prior to aging) demonstrated a marked increase in microhardness owing to precipitation strengthening and the improvement rate increases with increasing solid solution time. In addition, the microhardness of the post-cold-rolling samples is ~30 HV higher than that of their post-aging counterparts, which is attributed to the 80% cold-rolling.

**Figure 8:**
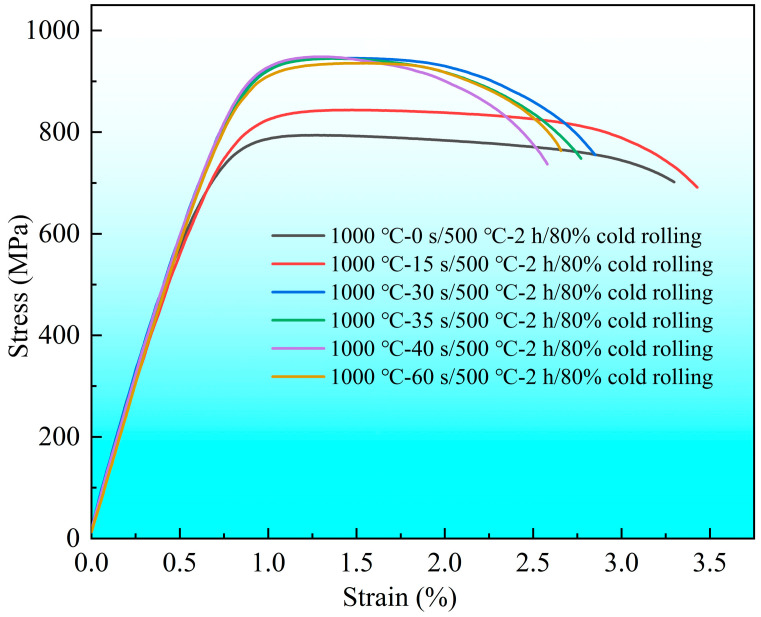
Tensile stress–strain curves of the Cu-Ni-Si-Mg-Mn specimens solid solution-treated at 1000 °C for varying durations, followed by aging at 500 °C for 2 h and a subsequent 80% cold-rolling.

**Figure 9:**
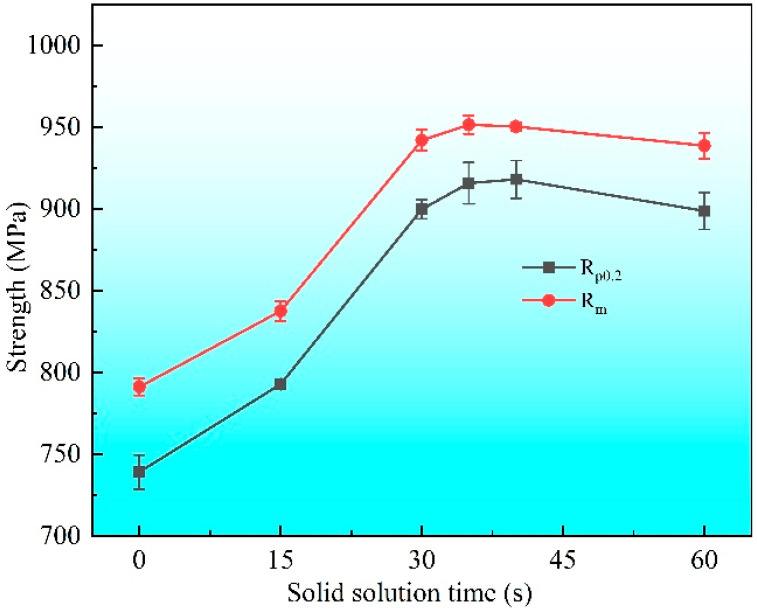
Evolutions in yield strength and tensile strength of the Cu-Ni-Si-Mg-Mn specimens solid solution-treated at 1000 °C for varying durations, followed by aging at 500 °C for 2 h and a subsequent 80% cold-rolling.

Figure 8 illustrates the tensile stress–strain curves of the specimens after solid solution, aging, and cold-rolling, and the corresponding yield strength and tensile strength are presented in Figure 9. As shown in Figure 8, the specimens without solid solution exhibit the lowest strength, while the specimen solution-treated for 15 s exhibits an obvious increase in strength and the highest elongation. For the other four groups of specimens, the tensile curves are relatively close, yet the elongation decreases with increasing solid solution time. Both the yield strength and tensile strength of the specimens first increase as the solid solution time increases, reaching peak values at a solid solution time of 35 s, as shown in Figure 9, and further prolonging the solid solution time results in a decline in strength. Although extending the solid solution time can increase the decomposition of residual precipitates, this increase cannot offset the decrease in strength caused by marked grain coarsening, thus both the strength and elongation decrease.

**Figure 10:**
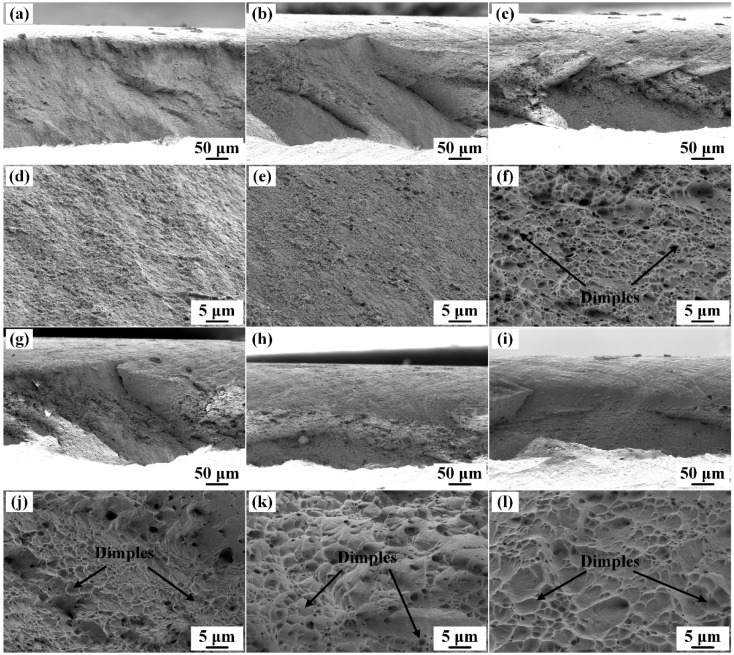
Tensile fracture surfaces of the Cu-Ni-Si-Mg-Mn specimens solid solution-treated at 1000 °C for varying durations, followed by aging at 500 °C for 2 h and a subsequent 80% cold-rolling; solid solution time: (**a**,**d**) 0 s, (**b**,**e**) 15 s, (**c**,**f**) 30 s, (**g**,**j**) 35 s, (**h**,**k**) 40 s, and (**i**,**l**) 60 s.

The tensile fracture surfaces of the specimens are presented in Figure 10, including both the macroscopic images and high-magnification morphologies at the center of the fracture surfaces. Although the elongation of all the specimens is quite low owing to cold rolling-induced hardening, obvious differences exist in fracture morphologies. Owing to the low elongation of the cold-rolled specimens, no obvious difference in necking was observed in the macroscopic fracture images. However, the high-magnification images reveal distinct differences in dimple features. Remarkably, the dimple structures are less obvious or very small for the non-solution-treated samples and the solid solution-treated for 15 s, although the elongations of the two groups of specimens are higher, probably because the negative effects of the cold-rolling before the solid solution are not completely eliminated during the solid solution treatment, and the very fine grain size restricts the formation of dimples. Once the solid solution time exceeds 30 s, the dimples become clear because the grain size is no longer so fine, and the size of the dimples increases with increasing solid solution time.

### 3.3. Evolution of Electrical Conductivity

**Figure 11:**
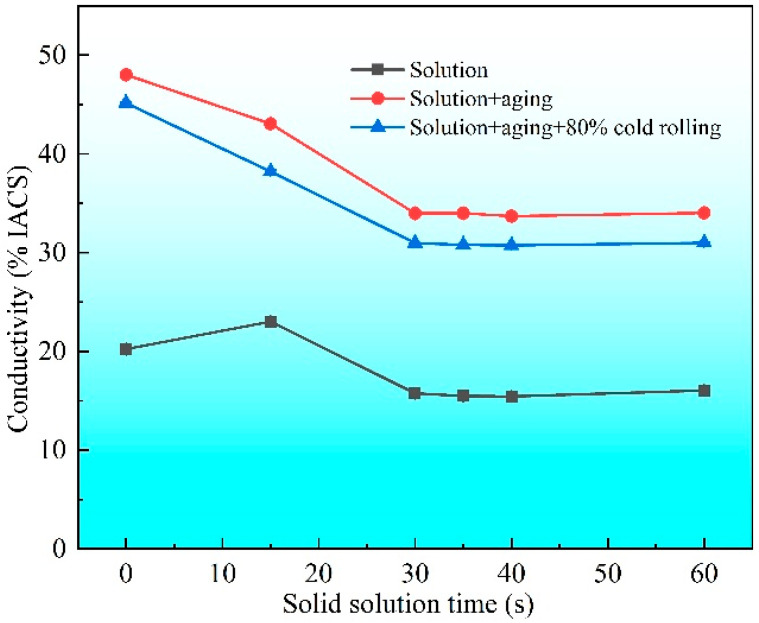
Variation in electrical conductivity with solid solution time for the specimens of the post-solution state, post-solution + aging state, and post-solution + aging + cold-rolling state.

For the application in BTB connectors, electrical conductivity is a crucial factor. Figure 11 shows the variation in electrical conductivity with solid solution time for the three sample conditions (solid solution-treated, post-aging, and post-cold-rolling). The electrical conductivity of a Cu alloy is determined by two key factors: the degree of solid solution and the density of defects. Following solid solution for 15 s, there was a slight increase in electrical conductivity, which indicates a remarkable reduction in deformation defects (e.g., dislocations from prior cold rolling), while precipitate decomposition and dissolution of the alloy elements is insignificant. As the solid solution time is further extended, additional alloy elements dissolve into the Cu matrix, resulting in a noticeable decrease in electrical conductivity. Once the precipitates are sufficiently decomposed and dissolved, the continued elimination of residual defects and the reduction in grain boundary density slightly increase the conductivity. Following aging, the electrical conductivity of all the specimens markedly increased owing to the elimination of defects and purification of the Cu matrix, resulting from precipitation, i.e., the precipitation of solute atoms (primarily Ni and Si) from the supersaturated solid solution. Notably, the highest conductivity is observed in the specimen aged without prior solid solution treatment, because most of the solute atoms remain undissolved in the matrix, and there is further precipitation during the aging process, leaving fewer solute atoms in the Cu matrix. With increasing solid solution time, additional alloy atoms dissolve into the matrix, and more residual solute atoms remain in the matrix even after aging, resulting in relatively lower conductivity. In addition, a subsequent 80% cold-rolling causes a 3% decrease in electrical conductivity compared with the aged specimens, which is attributed to the deformation defects introduced during cold-rolling.

## 4. Discussion

**Figure 12:**
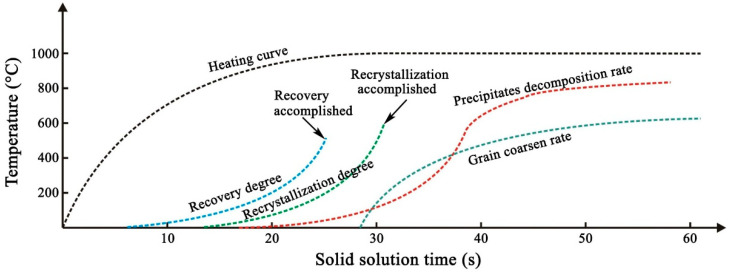
Semi-quantitative illustration of microstructure evolution of the cold-rolled Cu-Ni-Si-Mg-Mn high-solute alloy during solid solution.

Based on the results above, Figure 12 shows a semi-quantitative illustration of the microstructure evolutions of the cold-rolled Cu-Ni-Si-Mg-Mn alloy strip during the solid solution process. For the Cu-Ni-Si-Mg-Mn alloy, it can be predicted that the annihilation of the high-density dislocations would occur first during the solid solution process at 1000 °C, thus the microhardness decreases sharply. Following solid solution-treated at 1000 °C for 15 s, little precipitates decompose, thus the electrical conductivity even increases. Recrystallization started before precipitate decomposition, and it was completed after 30 s of solid solution, while sufficient decomposition of the precipitates requires at least 5 more seconds. Consequently, there is substantial grain coarsening with a slightly more decomposition of the residual precipitation particles.

Following the same aging and further cold-rolling, the influences of the solid solution time exist. The specimen solid solution-treated for 35 s exhibits the highest strength, although the decomposition of the precipitates is less than that subjected to a longer solid solution time, and the microhardness of the specimen solid solution-treated for 40 s is higher because the precipitation decomposition and aging precipitation are more sufficient. Four factors affect the strength, namely, the solid solution, grain size, precipitates, and the cold-working, and the overall strength of the alloy is dominated by their combined effects. For the Cu-Ni-Si-Mg-Mn alloy, it appears that the crucial solid solution period is 30–40 s, and it is affected by the thickness of the alloy strip, because both precipitate decomposition and grain coarsening occur severely during this period, and the decrease in strength caused by grain coarsening overcomes the contribution of precipitation strengthening, thus the strength increases to a peak and then decreases. Compared with the Cu-Ni-Si and Cu-Ni-Co-Si series alloys [30], sufficient decomposition of the precipitates is harder in the Cu-Ni-Si-Mg-Mn alloy. Therefore, a longer solid solution time or a slightly higher solution temperature should be required; while a tensile strength of >950 MPa has been achieved in this study, more precise control of the solid solution and aging temperature/time, and a combination of cold-rolling and aging are being explored.

## 5. Conclusions

This study systematically investigated the effects of short-time high-temperature solid solution, aging, and further cold-rolling on the microstructure and properties of the cold-rolled Cu-Ni-Si-Mg-Mn multi-element high-solute alloy. On the basis of the experimental results and discussion, the main conclusions are summarized as follows:

1. High-density precipitates, cold-working defects, and obvious deformation textures exist in the cold-rolled Cu-Ni-Si-Mg-Mn alloy strip. During the solid solution at 1000 °C, recrystallization occurs primarily between 15 and 30 s, when the alloy strip is heated, while precipitate decomposition starts at 30 s and almost completes 10 s later. A further increase in the solid solution time results in notable grain coarsening with little change in the residual precipitates.
2. Following solid solution treatment, aging, and 80% cold-rolling, a peak tensile strength exceeding 950 MPa and an electrical conductivity over 30% IACS were obtained in the strip solid solution-treated for 35 s. A further increase in the solid solution time decreases both the strength and elongation. The variation trend of microhardness is basically consistent with that of the strength, while the electrical conductivity almost stabilizes after solution treatment for over 30 s.
3. For the Cu-Ni-Si-Mg-Mn alloy, the crucial solid solution period is 30–40 s, and it will be affected by the thickness of the alloy strip. The main recrystallization and precipitate decomposition processes occur intensively within a few seconds. The solid solution temperature rather than time is the dominant factor for precipitate decomposition.

## Data Availability

The original contributions presented in this study are included in the article. Further inquiries can be directed to the corresponding author.
